# Predictive Value of TMJ Tomographic Parameters for Hypermobility‐Related Outcomes and Mouth Opening Capacity

**DOI:** 10.1111/joor.70194

**Published:** 2026-03-29

**Authors:** Samilla Pontes Braga, Rafaela Stocker Salbego, Camila Alves Carneiro, Maria Emilia Servin Berden, Carolina Ortigosa Cunha, Leonardo Rigoldi Bonjardim, Paulo César Rodrigues Conti

**Affiliations:** ^1^ Bauru School of Dentistry, Department of Prosthodontics and Periodontology, Bauru Orofacial Pain Group University of São Paulo Bauru São Paulo Brazil; ^2^ Bauru School of Dentistry, Department of Biological Sciences, Bauru Orofacial Pain Group University of São Paulo Bauru Brazil

**Keywords:** articular, joint dislocations, joint instability, range of motion, temporomandibular joint, temporomandibular joint disorders

## Abstract

**Background:**

Temporomandibular joint (TMJ) hypermobility may be asymptomatic or may progress to hypermobility disorders, in which excessive condylar translation predisposes to clinical manifestations such as subluxation or luxation, including open‐locking episodes. Although imaging provides structural parameters, its diagnostic value remains uncertain due to anatomical variability and inconsistent associations with symptoms.

**Objectives:**

To evaluate the predictive value of tomographic features for TMJ hypermobility disorders and mouth‐opening capacity.

**Methods:**

This analytical cross‐sectional study included 150 adults (18–40 years) divided into control, dysfunction, painful and combined TMD groups, diagnosed using standardised criteria. Clinical outcomes included mouth‐opening measures, open‐locking history and TMJ subluxation diagnosis. Cone beam computed tomography (CBCT) measures comprised condylar angle, anterior and superior distances of the condyle and Boering classification. Imaging followed standardised protocols. Regression analyses were performed with age, sex, condylar angle, superior distance of the condyle and Boering classification as predictors, after verifying model assumptions.

**Results:**

CBCT variables showed bilateral symmetry and strong intercorrelations; only right‐sided measures were retained. Multiple linear regression indicated that male sex predicted greater mouth opening across all measures, and superior distances of the condyle predicted greater assisted mouth opening. Binary logistic regression showed that older age reduced the odds of TMJ subluxation. Other imaging parameters were not significant, and the model for open‐locking history lacked explanatory power.

**Conclusion:**

Male sex was consistently associated with greater mouth opening, while imaging predictors showed only limited associations with functional measures. Age appeared protective against TMJ subluxation. CBCT parameters demonstrated limited predictive value for TMJ hypermobility‐related outcomes.

## Graphical abstract

Tomographic TMJ parameters showed limited predictive value for hypermobility‐related outcomes. Male sex was consistently associated with greater mouth opening, while increased age reduced the odds of TMJ subluxation. Structural imaging features demonstrated weak associations with functional measures, reinforcing the multifactorial nature of TMJ hypermobility and mandibular opening capacity.

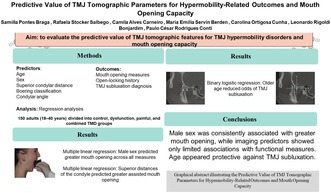

## 1 Background

Among temporomandibular disorders (TMD), TMJ hypermobility disorders are of particular clinical relevance. They are characterised by excessive condylar translation that exceeds the expected physiological range. In a subset of individuals, this increased translation may be associated with episodes of TMJ subluxation or luxation, rather than representing an inevitable or direct consequence of hypermobility [1, 2]. Accurate diagnosis is essential for appropriate management, yet current diagnostic criteria, such as the Diagnostic Criteria for Temporomandibular Disorders (DC/TMD), primarily rely on recent symptom history, which may not capture subclinical or remote manifestations of TMJ hypermobility [3].

Imaging examinations play a fundamental role in the assessment of TMJ morphology and function, providing information that complements the clinical examination [4]. These modalities allow the quantification of structural parameters such as the anterior and superior condylar distances in relation to the eminence and the degree of condylar translation relative to the articular eminence, classified by Boering's five‐point scale. This classification has been frequently cited as an auxiliary parameter for identifying TMJ hypermobility, with higher grades indicating greater anterior condylar translation [5, 6].

Despite these associations, the clinical utility of imaging findings remains uncertain, particularly regarding their ability to distinguish patients who experience clinical consequences of TMJ hypermobility, such as open‐locking episodes, from those in whom the condition is entirely asymptomatic. Moreover, these parameters do not account for normal anatomical variation, which may further limit their diagnostic specificity. In line with this concern, Kalaykova et al. (2006) demonstrated that although patients with clinical signs of TMJ hypermobility tended to present condyles positioned beyond the articular eminence at maximum mouth opening, a substantial proportion of asymptomatic individuals exhibited the same feature, indicating that condylar position alone is not a reliable predictor of clinical signs of TMJ hypermobility [7].

In addition to the uncertainty regarding the accuracy of imaging examinations in the context of TMJ hypermobility, there is also the widespread use of various terms such as TMJ subluxation, hypertranslation and hypermobility to describe what appears to be the same condition, a point highlighted and observed in a recent scoping review on the subject. While TMJ hypertranslation and hypermobility are often used interchangeably and largely describe an articular/kinematic feature (i.e., increased condylar excursion beyond the articular eminence), TMJ subluxation refers to a dysfunctional clinical phenomenon—typically reflecting symptomatic instability with clinically relevant outcomes (e.g., open‐locking episodes, functional limitation and pain)—and can therefore be interpreted as a manifestation or outcome associated with TMJ hypermobility rather than a purely anatomical trait. Another issue raised in the review was the high methodological variability in the assessment of TMJ hypermobility, ranging from clinical and anamnestic criteria to imaging‐based approaches [8].

Several references, including TMD and orofacial pain textbooks, TMJ anatomy references and scientific articles, have cited anterior positioning of the condyle relative to the articular eminence as a complementary finding in the diagnosis of TMJ hypermobility and/or subluxation [3, 5, 6, 9]. However, to date, few investigations have assessed the predictive capacity of specific tomographic measurements for hypermobility‐related disorders and individual mouth‐opening capacity. As a result, the potential role of imaging in improving diagnostic precision, identifying at‐risk individuals and guiding targeted management strategies remains unclear. Therefore, the objective of this study was to evaluate the predictive value of TMJ tomographic features for TMJ hypermobility disorders and mouth‐opening capacity.

## 2 Metodology

### 2.1 Ethics Statement

The study was approved by the Research Ethics Committee of the Bauru School of Dentistry, University of São Paulo (CAAE: 64579222.0.0000.5417; Approval No.: 6.296.473) and conducted in accordance with the principles of the Declaration of Helsinki. Reporting of this study followed the recommendations of the Strengthening the Reporting of Observational Studies in Epidemiology (STROBE) guidelines [10].

This was an analytical, cross‐sectional observational study conducted at the Bauru School of Dentistry, University of São Paulo, Bauru, São Paulo, Brazil, between September 2023 and July 2025. The target population consisted of adults aged between 18 and 40 years. All participants were informed about the study objectives and procedures and provided written informed consent prior to enrollment. The participants were 150 adults aged between 18 and 40 years. According to the DC/TMD Axis I framework, TMD can be categorised into pain‐related disorders and intra‐articular disorders [3]. Pain‐related TMD includes conditions such as myalgia, arthralgia and headache attributed to TMD, whereas intra‐articular TMD comprises disc displacement, degenerative joint disease and subluxation. This distinction has been applied in epidemiological and clinical studies, allowing for separate analyses of pain‐related and joint‐related conditions [11]. The sample (*N* = 150) was divided into four groups: a control group without TMD diagnosis and three TMD subgroups classified as dysfunction, painful and combined. The ‘painful TMD’ group comprised participants who met only the diagnostic criteria for pain‐related TMD, the ‘dysfunction TMD’ group included those diagnosed exclusively with intra‐articular disorders (subluxation and disc displacement with reduction with or without intermittent locking), and the ‘combined TMD’ group consisted of individuals meeting diagnostic criteria for both categories simultaneously. In addition, participants reported their history of open‐locking episodes, categorised as never, once in a lifetime, once per year, once per month or more than once per month.

Exclusion criteria included previous TMJ surgery, facial trauma, systemic conditions affecting joint mobility (e.g., heritable connective tissue disorders), current orthodontic treatment, TMJ degenerative joint disease and a diagnosis of disc displacement without reduction. TMJ degenerative joint disease and disc displacement without reduction were excluded to maintain a more homogeneous sample, as these conditions can reflect advanced stages of TMJ structural deterioration and functional limitation that differ substantially from less severe TMD presentations.

### 2.2 Imaging Protocol and Tomographic Measurements

All participants underwent bilateral TMJ imaging using cone‐beam computed tomography (CBCT) with a 3D Accuitomo XYZ Slice View Tomograph (J. Morita MFG. Corp., Kyoto, Japan) at the Radiology and Stomatology Department of the Bauru School of Dentistry, University of São Paulo. Images were acquired with participants in maximum mouth opening position, maintained with the aid of a customised bite opener, in an upright position with the Frankfort horizontal plane parallel to the floor. Acquisition parameters were: field of view (FOV) = 140 × 100 mm, tube voltage = 90 kV, tube current = 5.0 mA, scan time = 17.5 s, CTDIvol = 8.18 mGy, rotation = 360° and image mode = Standard.

Tomographic datasets were processed and analysed using the I‐Dixel imaging software (J. Morita MFG. Corp., Kyoto, Japan)—3DXD Version 5.0.3.13757/DixelD Version 7.3.2.0—in conjunction with the 3DX Integrated Information System (version 2.4.0.3). This platform enables high‐resolution multiplanar reconstruction, orientation of slices perpendicular to the condylar long axis and precise linear and angular measurements using standardised anatomical landmarks. For each TMJ, the parasagittal slice that best depicted the condyle‐articular eminence relationship was selected for measurement.

**FIGURE 1:**
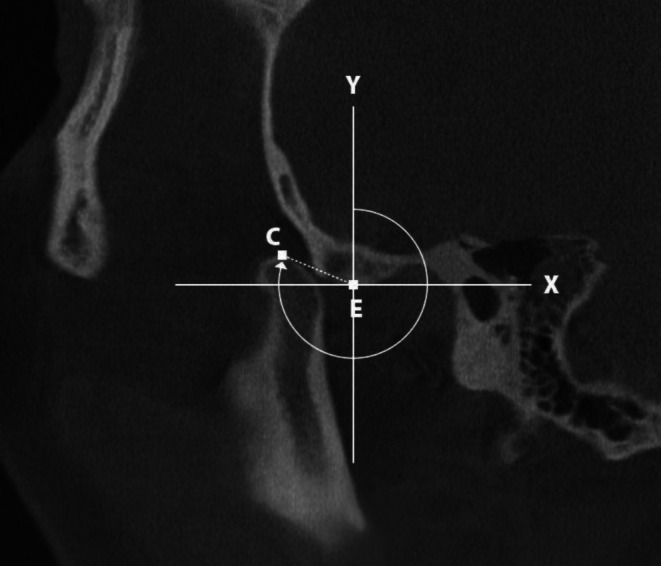
Measurement of the condylar angle (°) according to the method of Kalaykova et al. (2006), adapted for CBCT images. In the parasagittal slice, point E corresponds to the most inferior aspect of the articular eminence crest and point C corresponds to the most superior aspect of the condylar cortex. A vertical line (Y‐axis) and its perpendicular horizontal line (X‐axis) were drawn from point E to establish a Cartesian coordinate system. The condylar angle was defined as the angle between the EC line and the vertical axis.

Condylar angle (°) (Figure 1): The condylar angle was measured following the method described by Kalaykova et al. (2006), adapted for CBCT images. In the parasagittal slice that best displayed the condyle‐articular eminence relationship, two anatomical landmarks were identified: E (eminence point): the most inferior point of the articular eminence crest; C (condylar point): the most superior point of the condylar cortex. From point E, a vertical line (Y‐axis) and a horizontal line (X‐axis) perpendicular to it were constructed, establishing a Cartesian coordinate system. The condylar angle was defined as the angle between the line connecting points E and C (EC) and the vertical Y‐axis. Larger angles indicate more anterior and/or superior condylar positioning relative to the eminence crest, with values exceeding 180° representing positions anterior to the crest [7].

**FIGURE 2:**
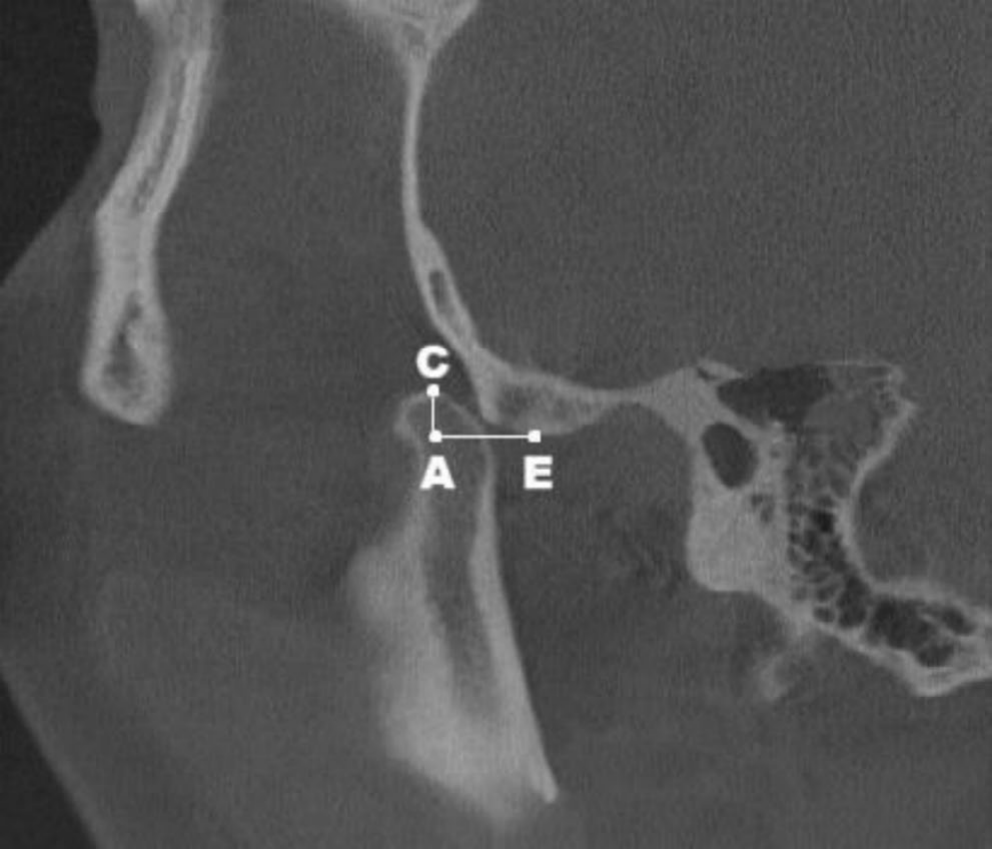
Measurement of the anterior and superior condylar distances to the eminence (mm), using the same parasagittal slice and Cartesian reference system defined for the condylar angle. The anterior condylar distance was measured as the shortest linear distance from point E (eminence point) to point A (central point of the condyle) along the X‐axis, representing the anterior‐posterior condylar position. The superior condylar distance was defined as the linear distance from point A to point C (most superior point of the condylar cortex) along the Y‐axis, representing the vertical condylar relationship to the eminence crest.

Anterior condylar distance to the eminence (mm) (Figure 2): Using the same parasagittal slice and reference system defined for the condylar angle, the anterior condylar distance was measured as the shortest linear distance, in millimetres, from point E (eminence point) to the most central point of the condyle (Point A), projected along the horizontal X‐axis. This measurement represents the anterior‐posterior positioning of the condyle relative to the eminence. Superior condylar distance to the eminence (mm) (Figure 2): In the same coordinate system, the superior condylar distance was defined as the linear distance, in millimetres, from the most central point of the condyle (point A) until point C (the most superior point of the condylar cortex), measured along the vertical Y‐axis. This parameter quantifies the condyle's vertical relationship to the eminence crest [7].

Boering classification (grades 0 until 4): Condylar translation relative to the articular eminence during maximal unassisted mouth opening was categorised according to the Boering classification as follows: Grade 0: The condyle does not translate, or translates only slightly anteriorly (severely restricted mobility). Grade 1: The condyle translates anteriorly but does not reach the top of the articular eminence (restricted mobility). Grade 2: The condyle translates to the top of the articular eminence (normal mobility). Grade 3: The condyle translates over the top of the articular eminence (increased mobility, still within normal limits but tending towards hypermobility). Grade 4: The condyle translates excessively, well beyond and superior to the level of the top of the articular eminence (hypermobility) [5, 6].

All tomographic measurements were performed by a single examiner experienced in TMJ imaging analysis, blinded to clinical information (DC/TMD diagnoses and patient‐reported symptoms). Blinding was maintained by assigning numeric study IDs and withholding access to clinical records during image review. For each TMJ, linear (anterior and superior condylar distances) and angular (condylar angle) measurements were obtained in duplicate to verify stability. When repeated values differed by > 0.5 mm (linear) or > 1° (angular), the measurement was repeated until two consecutive readings met these thresholds; the final recorded value was the stable reading. The Boering classification was assigned once per TMJ according to established criteria. All measurements were performed using the I‐Dixel software (J. Morita MFG. CORP., Kyoto, Japan).

### 2.3 Clinical Outcomes

All TMD diagnoses were established following the standardised DC/TMD Axis I protocol. Mouth opening was assessed following the standardised procedures of the DC/TMD protocol using a conventional millimetre ruler. Two measures were obtained: unassisted maximum mouth opening (maximum opening regardless of pain), and assisted maximum mouth opening (the examiner applied gentle additional pressure with the fingers on the incisors, stopping immediately if the participant signalled discomfort). In both measures, the measurement corresponded to the distance between the incisal edges of the maxillary and mandibular central incisors, plus the overbite value when present [3].

### 2.4 Statistical Analysis

The sample size calculation considered up to four main predictors (condylar angle, anterior condylar distance, superior condylar distance and Boering classification) which represented the central tomographic variables of interest. The calculation, performed for multiple regression with up to four independent variables, assuming α = 0.05 and 80% power, indicated a minimum required sample of *N* = 108 participants. The final sample comprised *N* = 150, thus exceeding the requirement and ensuring adequate statistical power.

Right‐left comparisons of tomographic measurements (condylar angle, anterior distance and superior distance) were performed using the non‐parametric Wilcoxon signed‐rank test, as preliminary normality tests (Shapiro‐Wilk, Kolmogorov‐Smirnov and Anderson‐Darling) indicated that at least two of the variables did not meet the assumption of normally distributed differences. And to assess potential collinearity among the imaging predictors, a preliminary Pearson correlation analysis was performed. Based on these preliminary analyses, some variables were excluded to reduce redundancy. Thus, the predictors analysed in the regression models were: age, sex, superior condylar distance, Boering classification and condylar angle, all on the right side. Before performing the regression analyses, the assumptions of the models were verified, including normality of residuals, homoscedasticity, absence of autocorrelation and multicollinearity.

The dependent variables included: unassisted maximum mouth opening, assisted maximum mouth opening (all in millimetres), open‐locking history (never; once in a lifetime; once per year; once per month; more than once per month) and TMJ subluxation diagnosis according to DC/TMD (yes/no).

## 3 Results

The sample (*N* = 150) was predominantly female (71.3%); the mean age was 27.39 ± 5.67 years and consisted mainly of individuals with some TMD diagnosis (84.0%). Among the 150 participants, 24 (16.0%) were classified as healthy controls, 28 (18.7%) as dysfunction TMD, 26 (17.3%) as painful TMD and 72 (48.0%) as combined TMD—indicating that nearly half of the sample fell into the combined subtype, while the other three categories were smaller and of similar magnitude. A DC/TMD diagnosis of TMJ subluxation was present in 16.7%. Disc displacement with reduction (DDWR) was observed in 62.0% (unilateral 48.0%; bilateral 14.0%), and DDWR with intermittent locking in 16.7%. Regarding open‐locking history, 54.7% reported never experiencing it, 21.3% a single lifetime episode, 5.3% once per year, 9.3% once per month and 9.3% more than once per month.

The Wilcoxon signed‐rank test showed no significant differences between the right and left sides for any of the tomographic measures: condylar angle (W = 4926, *p* = 0.760), anterior distance to the eminence (W = 5211, *p* = 0.986) and superior distance to the eminence (W = 4187, *p* = 0.457). These findings confirm bilateral symmetry and low lateral variability. The Pearson correlation analysis showed strong correlations among the tomographic measures, as homologous right‐left variables (*r* = 0.854), so all measurements related to the left TMJ were removed to minimise redundancy in models. Also, linear measures of anterior and superior distances (up to *r* = 0.826), and between condylar angles and anterior distances on the same side (approximately *r* = 0.80) were strongly correlated; thus, the anterior distance to the eminence measurement was excluded from the regression models too. The predictors analysed in the regression models were: age, sex, superior condylar distance, Boering classification and condylar angle, all on the right side.

A multiple linear regression analysis was conducted with unassisted maximum mouth opening as the dependent variable (Table 1). The model met all assumptions, with normally distributed residuals, homoscedasticity, no autocorrelation (Durbin–Watson = 1.88, *p* = 0.436) and VIFs below 3. Among the predictors, only sex (*β* = −5.57, *p* < 0.001) was significant, indicating that women had smaller unassisted maximum mouth opening. All other predictors were not significant (*p* > 0.05). Another multiple linear regression analysis was also performed using assisted maximum mouth opening as the dependent variable (Table 2). The model met all assumptions, with normally distributed residuals, homoscedasticity, no autocorrelation (Durbin–Watson = 1.80, *p* = 0.186) and VIFs below 3. Two predictors showed significant associations: superior distance to the eminence (*β* = 0.90, *p* = 0.014), indicating that greater values were associated with larger assisted maximum mouth opening, and sex (*β* = −4.30, *p* < 0.001), with women presenting smaller values of the outcome. All other predictors were not significant (*p* > 0.05).

**TABLE 1:** Multiple linear regression model with *N* = 150, *R* ^2^ = 0.282, adjusted R^2^ = 0.252, *p* < 0.001.

Predictor	Estimatives	Standard error	*p*	Standardised estimates	Confidence interval (95%)	
					Lower limit	Upper limit
Intercept	49.1493	8.6132	< 0.001			
Age	−0.0190	0.0948	0.841	−0.0145	−0.15732	0.128
Superior distance (mm)	0.7977	0.4079	0.052	0.2324	−0.00252	0.467
Boering class						
3–2	3.0419	6.6507	0.648	0.4064	−1.35004	2.163
4–2	4.6954	7.3869	0.526	0.6272	−1.32357	2.578
Sex Female (ref: Male)	−5.5670	1.2122	< 0.001	−0.7437	−1.06380	−0.424
Condylar angle	0.0188	0.0513	0.714	0.0734	−0.32174	0.469

**TABLE 2:** Multiple linear regression model with *N* = 150, *R* ^2^ = 0.326, adjusted *R* ^2^ = 0.297, *p* < 0.001.

Predictor	Estimatives	Standard error	*p*	standardised estimates	Confidence interval (95%)	
					Lower limit	Upper limit
Intercept	48.5919	7.6774	< 0.001			
Age	−0.0415	0.0845	0.624	−0.0344	−0.1728	0.104
Superior distance (mm)	0.9035	0.3635	0.014	0.2862	0.0586	0.514
Boering class						
3–2	2.0763	5.9281	0.727	0.3016	−1.4005	2.004
4–2	3.3660	6.5843	0.610	0.4889	−1.4016	2.379
Sex Female (ref: Male)	−4.3011	1.0805	< 0.001	−0.6247	−0.9349	−0.314
Condylar angle	0.0348	0.0457	0.447	0.1476	−0.2353	0.531

A binary logistic regression using TMJ subluxation diagnosis (no/yes) as the dependent variable was performed (Table 3). The assumptions of the binary logistic regression were verified. No evidence of relevant multicollinearity was found among predictors (all VIF < 2.5). Observations were independent, and no violations of the linearity assumption were detected. The global test showed a significant overall model fit (*χ* ^2^ = 17.2, gl = 6, *p* = 0.009; *R* ^2^ = 0,187). In the omnibus test, only age was significantly associated with the outcome (*p* = 0.001), with higher age values linked to lower odds of TMJ subluxation diagnosis (OR = 0.868; 95% CI = 0.788–0.956; *p* = 0.004), suggesting a protective effect. All other predictors were not significant (*p* > 0.05).

**TABLE 3:** Binomial logistic regression model with *N* = 150, *χ* ^2^ = 17.2; gl = 6; *p* = 0.009; *R* ^2^ = 0.187.

						Confidence interval (95%)	
Predictor	Estimatives	Standard error	*z*	*p*	Odds ratio	Lower limit	Upper limit
Age	−0.1421	0.0493	−2.87995	0.004	0.868	0.788	0.956
Superior distance (mm)	0.1932	0.2107	0.91709	0.359	1.213	0.803	1.834
Boering class							
3–2	14.2772	1694.2137	0.00843	0.993	1.59e+6	NE	NE
4–2	14.2819	1694.2155	0.00843	0.993	1.59e+6	NE	NE
Sex Female (ref: Male)	−0.4454	0.5198	−0.85687	0.392	0.641	0.231	1.774
Condylar angle	0.0115	0.0496	0.23144	0.817	1.012	0.918	1.115

*Note:* Values expressed in scientific notation (e.g., 1.59e+6) indicate unstable estimates due to sparse data and should not be interpreted clinically.

Abbreviation: NE, not estimable.

A multinomial logistic regression was performed with open‐locking history as the dependent variable (never; once in a lifetime; once a year; once a month; more than once a month) using the same predictors. The global test (*χ* ^2^ = 33.5; gl = 24; *p* = 0.093; *R* ^2^ = 0.112) was not statistically significant, indicating that the set of predictors did not explain the outcome with sufficient robustness. Therefore, this analysis was not retained in the final results.

## 4 Discussion

According to the American Academy of Orofacial Pain (AAOP), the normal jaw opening ranges between 40 and 55 mm [1, 12]. Although these are generally accepted ranges, individual measurements may vary depending on many factors, such as height and craniofacial shape. Several studies report that the normal opening range is smaller in women than in men and decreases with increasing age [13, 14, 15]. Among the results, sex was the most consistent predictor: the female sex was associated with smaller mouth opening across all outcomes: −5.57 mm for unassisted maximum opening (*p* < 0.001) and −4.30 mm for assisted maximum opening (*p* < 0.001). Among the other predictors, the superior distance to the eminence was positively associated with assisted maximum opening (*β* = 0.90; *p* = 0.014); all remaining effects were not significant.

Previous studies that assessed the influence of sex on mandibular structures or other structures of the craniomaxillofacial complex found that measurements in males are greater than in females [16, 17]. However, multiple anatomical factors influence TMJ morphology and mouth‐opening biomechanics. In a CBCT study of Class II individuals, facial growth pattern, rather than sagittal Class II relationship, was the main determinant of TMJ morphology [18]. Collectively, kinematic studies in healthy individuals demonstrate that maximum mouth opening does not reliably reflect condylar translation, as similar opening amplitudes may result from different combinations of rotation and translation. Mandibular length and neuromuscular control appear to play a more relevant role than condylar displacement alone. However, as these investigations included only asymptomatic participants, their findings cannot be directly extrapolated to patients with TMD [19, 20, 21].

In a computed tomography (CT)‐based study, increasing mouth‐opening amplitude was associated with progressively greater anterior condylar displacement, ranging from a posterior position relative to the articular eminence in low hypermobility to an anterior position in marked hypermobility, despite methodological limitations [22]. In the present study, a greater superior condylar distance relative to the articular eminence predicted larger assisted mouth opening. This finding suggests that a more superior condylar position may facilitate increased opening amplitudes when mandibular movement is passively potentiated, reflecting a greater passive mechanical capacity of the condyle‐eminence complex. Importantly, the relationship between mouth opening and condylar displacement is neither linear nor universal, as it depends on the type of movement (active vs. assisted) and the morphofunctional profile analysed (normal vs. increased mobility). In contrast, unassisted mouth opening and the clinical manifestations of TMJ hypermobility appear to rely more on neuromuscular control and functional factors than on condylar position alone.

Regarding morphological factors associated with TMJ hypermobility, imaging studies suggest that hypermobility is frequently accompanied by radiographic degenerative changes, such as condylar flattening and subchondral sclerosis, but without consistent associations with pain or functional outcomes [5, 23, 24]. This pattern supports the interpretation of TMJ hypermobility as an articular phenotype characterised by structural remodelling rather than by clinically manifest dysfunction. However, the interpretability of these findings is limited by non‐standardised diagnostic criteria, small or heterogeneous samples and reliance on static or qualitative imaging methods. Consistent with this, CT evidence indicates that condylar morphology alone does not predispose to TMJ luxation [25]. In the present study, positional parameters were associated with greater mouth‐opening amplitude but showed limited ability to explain clinically relevant outcomes, reinforcing that joint morphology influences mandibular mechanics without independently determining TMJ subluxation or luxation.

One of the first studies to address the relationship between condylar position and signs of TMJ hypermobility showed that condyles beyond the eminence were common in patients with clinical signs of TMJ hypermobility, such as movement incoordination and terminal joint sounds, but also present in nearly half of controls [7]. Consistently, in the present sample, condylar position partially predicted increased mouth‐opening amplitude but failed to explain clinically relevant outcomes, such as open‐locking or TMJ subluxation. Complementary MRI and biomechanical studies further support that condylar hypertranslation is a necessary but not sufficient condition for TMJ luxation or subluxation [26, 27].

The biomechanical model proposed by Tuijt et al. (2018) demonstrated that TMJ instability arises when elevator muscles must compensate excessively to maintain condylar stability in extreme positions, indicating that condylar translation beyond the eminence alone is insufficient to predict dysfunction [27]. A complementary kinematic study showed that mouth‐opening trajectories vary widely among individuals, with unstable condylar paths often presenting as irregular closing patterns or condylar ‘jumps’ [21]. Together, these findings emphasise that neuromuscular coordination and muscular demand, rather than morphology alone, are central determinants of whether hypertranslation remains asymptomatic or progresses to clinical instability.

In this study, imaging predictors showed no significant explanatory value. This finding is consistent with the critical appraisal by Steenks et al. (2018), who questioned the diagnostic validity of the ‘subluxation’ category in the DC/TMD. The authors argued that the term has been inconsistently used in the literature, often generating confusion and that condylar translation beyond the articular eminence during wide opening is observed in the majority of the general population without symptoms or functional limitation [28]. In fact, Okeson (2020) describes TMJ subluxation as a non‐pathological anatomical variation, whereas the DC/TMD framework categorises it as a distinct TMD subtype [2, 3]. This conceptual divergence is not merely semantic but has direct implications for both research and clinical practice: classifying subluxation as a disorder risks pathologising a physiological variant commonly observed in the general population, while treating it as benign may overlook cases that evolve into recurrent luxation with functional impairment. This ongoing debate underscores the need for clearer diagnostic boundaries and evidence‐based criteria to determine when TMJ subluxation represents a clinical problem rather than an anatomical variation.

The fact is, reliance on imaging alone, such as the Boering classification of condylar position relative to the eminence, risks overdiagnosis, since these radiographic thresholds do not necessarily correlate with symptoms or functional impairment [5, 6]. Being the TMJ luxation and/or subluxation a potential consequence or an unfavourable clinical course of TMJ hypertranslation, the factors that determine which patients with TMJ hypermobility are asymptomatic and which will develop TMJ luxation or subluxation are still unclear. Clearly, there are predisposing and modulating factors involved.

Among the morphological or structural factors mentioned, the morphology of the articular eminence has been linked to TMJ luxation, with flat or low eminences favouring condylar displacement beyond the eminence, while steep eminences provide greater containment. Although early clinical and radiographic studies supported this association [5, 6], more recent imaging analyses suggest that eminence morphology alone shows only a limited correlation with actual TMJ luxation events [27]. While the TMJ morphology, such as eminence slope, contributes, predictive accuracy improves only when factors like ligament integrity and neuromuscular control are incorporated. A previously proposed TMJ hypermobility model suggests that instability arises when muscular compensation is needed to stabilise the condyle at extreme positions, underscoring the importance of combining clinical, imaging and subjective markers for diagnosing TMJ hypermobility, despite its current theoretical nature and lack of large‐scale validation [27]. It should be noted that articular eminence angulation was not quantitatively assessed in the present study. Future investigations should explore the potential interaction between eminence inclination and condylar position to better clarify their combined influence on mouth‐opening capacity and TMJ hypermobility.

In the present analysis, age emerged as the only significant factor associated with TMJ subluxation, with younger individuals showing higher odds of diagnosis. This is consistent with the notion that joint hypermobility tends to manifest more prominently in younger patients, paralleling findings on disc displacement with reduction, described by Poluha et al. (2018), which also shows higher prevalence in younger women and often follows a benign natural course. In both conditions, clinical expression appears to be conditional rather than inevitable, with anatomical predispositions gaining significance only when combined with mechanical load and insufficient adaptive capacity [29].

## 5 Conclusions

It can be concluded that male sex was consistently associated with greater mouth opening, whereas the imaging parameter superior condylar distance showed only an association with assisted mouth opening. Age appeared to exert a protective effect against TMJ subluxation. In contrast, the regression model for open‐locking history did not reach statistical significance. Taken together, these findings indicate that the evaluated imaging parameters have limited predictive value for TMJ hypermobility‐related outcomes such as TMJ subluxation and open‐locking history. From a clinical standpoint, this suggests that dynamic functional and adaptive factors, rather than static anatomical measurements, may play a more decisive role in determining the manifestation and clinical course of TMJ hypermobility.

## Author Contributions

Samilla Pontes Braga (SPB) was responsible for study conception and design, data collection, data curation, statistical analysis, interpretation of the results and drafting of the manuscript. Rafaela Stocker Salbego (RSS) performed the statistical analysis and contributed to data interpretation. Camila Alves Carneiro (CAC), Maria Emilia Servin Berden (MESB), Carolina Ortigosa Cunha (COC), Leonardo Rigoldi Bonjardim (LRB) and Paulo César Rodrigues Conti (PCRC) critically revised the manuscript and approved the final version for publication. All authors read and approved the final manuscript.

## Funding

This work was supported by Coordenação de Aperfeiçoamento de Pessoal de Nível Superior, 88887.916070/2023‐00, 88887.885902/2023‐00.

## Conflicts of Interest

The authors declare no conflicts of interest.

## Data Availability

The data that support the findings of this study are available from the corresponding author upon reasonable request.
